# Editorial: Plants' Responses to Novel Environmental Pressures

**DOI:** 10.3389/fpls.2017.02000

**Published:** 2017-11-23

**Authors:** Alessio Fini, Massimiliano Tattini, Raquel Esteban

**Affiliations:** ^1^Department of Agricultural and Environmental Sciences —Production, Landscape, Agroenergy, University of Milan, Milan, Italy; ^2^Department of Biology, Agriculture and Food Sciences, National Research Council of Italy (CNR), Institute for Sustainable Plant Protection, Sesto Fiorentino, Italy; ^3^Department of Plant Biology and Ecology, University of the Basque Country (UPV/EHU), Bilbao, Spain

**Keywords:** adaptation, acclimation, drought, migration, multiple stress, pollution, warming

Multiple environmental stressors, on both short-term and long-term basis, challenged early land plants since they colonized the land, around 450 million years ago. Emerged lands were far more sensitive to changes in climate than oceans, and plants have displayed during evolution an extraordinary ability to survive (i.e., to adapt) dramatic variations in environmental conditions. This has occurred through acclimation, adaptation, or migration to new environments (Bussotti et al., 2014). Migration is a very long process, as plants do not possess the “flight” strategy displayed by other living organisms, and it is further hampered by environmental, biological, and spatial barriers (Bussotti et al., 2014). For example, high UV-load may hasten the upward migration of tropical alpine plants. Barnes et al., in this special issue, tested the hypothesis that more effective photoprotective mechanisms may explain the greater upward migration potential of exotic compared to native species. After surveying the leaf epidermal UV-A-transmittance (inversely related to the accumulation of flavonoids and related UV-absorbing compounds) in 54 species, the authors found that plasticity in this parameter poorly explained the different migration potential of native and exotic species, despite significant intra-specific differences. Rull and Vegas-Vilarrubia also present a remarkable case study of the impact of barriers on plant migration. Authors draw the attention on “The Lost World,” the neotropical Guayana highlands. This pristine habitat, which never suffered from direct human pressure, has been recently exposed to the indirect pressure of human activities, such as global warming and altered rainfall regime. Migration toward higher altitudes is prevented in this habitat because of the flat tops of the highlands. Species with higher phenotypic and physiological plasticity are better suited to survive the challenges associated to climate change. The relative significance of environmental/ and “spatial” (barrier) factors on vegetation dynamics has been explored, at large level of scale, by Chen et al. Authors give compelling evidence that environmental factors are primary drivers of shrub species turnover, whereas barriers mostly hamper the turnover of trees. Wang Z. et al. have also explored traits, such as the germination potential, responsible for effective species dispersal. It is shown that this trait is under tight control of environmental pressures, on short-term scale level, whereas phylogenic constrains become prevalent on long-term basis.

It is not surprising to observe that plants, due to their sessile nature and their slow migration speed, have developed a complex suite of modular and highly integrated mechanisms to match the key constrains encountered within the species distribution range, broadly the acclimation/adaptation syndrome (Lichtenthaler, 1998; Demmig-Adams et al., 2014; Esteban et al., 2015). The ability to adjust morpho-physiological and biochemical traits at different time scales (i.e., over seconds at the chloroplast and cellular level, or over days, growing seasons, or even generations at the whole-canopy level) constitutes the “flight strategy of sessile organisms” (Potters et al., 2007; Karban, 2008). Investigating three ecotypes of *Arabidopsis thaliana*, Adams et al. evidenced the relevant effects of native habitat on anatomical and functional traits of leaves. Interestingly, while the ecotype from the central portion of the distribution range showed the highest plasticity to environmental changes, accessions from the Northern and Southern portions showed highly distinct adaptive traits to cope with cool temperatures and drought, respectively. Similarly, to survive to the harsh Mediterranean climate, plants have developed a wide range of adaptive traits, including a fine tuning of phenological phases to maximize carbon gain, while avoiding frost damage in winter (Cleland et al., 2007), and specific morpho-physiological characteristics to cope with summer drought (Bussotti et al., 2014). Consistently, Fernandez-Marín et al. pointed out that photoprotective mechanisms of Mediterranean flora are highly adapted to the environmental pressures associated to Mediterranean climate, namely drought, excess light, and high temperature, with α-tocopherol and violaxanthin-cycle pigments being more responsive to such stresses in Mediterranean than in other flora. It is worth noting, that the priming driven by stressful conditions enable plants, not only animals, to acclimate successfully to recurrent stress episodes, i.e., living organisms have the capacity to remember stress events (Conrath et al., 2006), as pointed out, in this special issue, by Matsubara et al. The extraordinary capacity to acclimate and adapt to their growing environment is observed indeed at the individual scale, as well as at larger scales (e.g., cenosis and ecosystem): de la Riva et al. provided rationale of such biodiversity, and highlighted that different biogeochemical niche partitioning allows species to coexist through divergent leaf nutrient composition and resource uptake.

It is not surprising indeed that several habitats threatened by the most severe environmental pressures are biodiversity hotspots. For example, Mediterranean ecosystems, where plants are simultaneously challenged by high light, heat, and drought stress of increasing severity, represent just 2% of the earth's land area, but account for 16% of the world's plant species (Myers et al., 2000). Similarly, mountain ecosystems and nutrient-poor soils are biodiversity hotspots highly endangered by climate change (Lambers et al., 2013). Although land plants have long history of successful response to the challenges imposed by a changing environment, climate change in the Anthropocene has peculiarities, which can turn old stresses into something “novel” to plants.

First, the unprecedented rate at which climate changes may exceed the rate of genetic adaptation and migration in most species (Klein et al., 2013), with some functional groups being more affected than others. Exploring the effect of changes in night temperatures on different plant functional types, growth forms, and economic purposes (C3 vs. C4 vs. CAM; herbaceous vs. woody; crop vs. no-crop), Jing et al. highlighted that the large variation in the acclimation capacity of the different plant species may yield different fitness under future climate scenarios. Wang D. et al., evaluating long-term response to heat stress in C3 and C4 species, offered evidence that repeated, even transient heat stress episodes can affect plant community structure, vegetation dynamics, biodiversity, and ecosystem functioning on long-term basis. Interestingly, plasticity during stress acclimation does not only vary among functional groups, species, accessions and ecotypes, but also between male and female plants in dioecious species. The topic is explored by Jiang et al., who found important trade-offs between growth in male and female individuals of *Salix paraplexia* growing under poor nutrient availability, with females investing more in growth than males. The authors concluded that a moderate increase in female willow species in restoration programs could increase the resistance and resilience of willow populations to early sporadic desertification. Similarly, on the dioecious palm *Chamaerops humilis*, Morales et al. found that female individuals were more sensitive than males to winter photoinhibition. These results may be, therefore, helpful for restoration decisions focused on sex selection.

Secondly, unprecedented biotic and abiotic stress events, such as heat waves, drought spells, (introduced) pest outbreak, and human disturbance, often occur simultaneously and interact with each other. While literature is full of experiments evaluating plant response to single stressors, research investigating the interaction among co-occurring stressors is scarce (Dieleman et al., 2012). Trying to fill this gap, the research by Top et al. focused on the effects of high temperatures and water availability on secondary metabolites in leaves of *Quercus rubra*. In comparison to plants growing under favorable climate, the plants challenged by stress factors produced more tannins with lower degree of complexity, i.e., with reduced chain length. This may have relevant consequences on both carbon and nutrient recycling and, as consequence not only on the short-time “adaptation” of species to adverse environmental pressures associated to climate change, but also in the regulation of herbivore dynamics. How physiological and metabolic adjustments triggered by the acclimation to abiotic stresses affect plant response to pests is indeed intriguing. Ederli et al. investigated the influence of drought stress on *Nezara viridula* infestation and nymph growth performance in *Vicia faba*. Authors highlighted a role for salicylic acid signaling in both drought stress and *N. viridula* infestation, whereas ABA-signaling is either activated by drought or down-regulated by *N. viridula* infestation, thus offering new insights about how plants respond to concurrent abiotic and biotic stressors. The interaction effects of UV-B, light quality and quantity, heat and drought stresses, on plant defenses against herbivores also constitute the main issue explored in the review article by Escobar-Bravo et al. UV-B can interact positively with high photosynthetic active radiation, blue light, high temperature, and water deficit to increase plant performance and constitutive chemical defenses. Nonetheless, fresh assimilated carbon available to the synthesis of secondary metabolites may be severely constrained at severe heat and drought stress, and alternative carbon sources are required for their biosynthesis. It is suggested that UV-B radiation, possibly in combination with high blue light, may enhance plant defense against herbivores, especially in high density planting, as herbivores arthropods take advantage in far red-to-far red-enriched environments.

Besides host–pathogen interaction, the relationship between plants and beneficial microorganisms is highly sensitive to the novel pressures imposed by global change (Compant et al., 2010). For example, there is evidence that this beneficial interaction may be favored by rising CO_2_ and mild drought, but hastened by severe disturbances, such as urbanization (Fini et al., 2011). Understanding the plant-microbe relation under the climatic pressure associated to climate change, is crucial for deeper insights into stress-mitigation mechanisms. Meena et al. reviewed the abiotic stress responses and the microbe-mediated mitigation through multi-omics strategies. Authors offered an exhaustive view of the key roles of multi-omics approaches, such as transcriptomics, proteomics, metabolomics, and phenomics in providing a better understanding of the complex interaction of plant-microorganisms.

Thirdly, the release of gaseous, solid, and electromagnetic pollution from human activities has increased greatly since the industrial age, and constitutes a novel “oxidative” threat for plants. Primary pollutants, such as nitrogen oxides and carbon monoxide, are highly reactive and can either direct injury plants or combine in the troposphere into secondary pollution, a hard-to-solve issue for plant community health. The input of reactive nitrogen in the atmosphere has fostered nitrogen deposition and acid precipitation, with detrimental effects on biodiversity on global scale (Sala et al., 2000). Mao et al. investigated the effects on nitrogen addition and altered snowfall pattern at different scales, from the intra-specific variability of leaf traits to the functional diversity of the whole community. Authors found positive effect of nitrogen addition on the overall growth of plant communities, but highlighted that annual plants shifted faster than perennials to more nitrophilic functional traits (e.g., low leaf mass per area), which may determine a change of dominant species in plant communities. Ozone is a secondary gaseous pollutant markedly influencing plant communities. Cotrozzi et al. explored the interaction between ozone and drought stresses on the evergreen broad-leafed *Quercus ilex*. It is shown, that plants challenged by water deficit display decreased capacity to activate phytohormone-related signaling pathways, and hence proper warning signals, when successively exposed to acute O_3_ stress. Ozone is formed in the troposphere when nitrogen oxide reacts with volatile organic compounds (VOC). A fraction of total VOCs in the atmosphere has biogenic origin (BVOC, mainly isoprene and monoterpenes) and primarily emitted by plants. After reviewing why biogenic isoprene emission may become relevant in a warmer and drier climate, Fini et al. focused on its metabolic roles, with particular reference to hygrophilic species. Most isoprene emitters are hygrophylic and display a constitutive suite of morpho-anatomical and physiological features unable to efficiently counter the challenges imposed by harsh climates (Valladares et al., 2000; Tattini et al., 2006; Matesanz and Valladares, 2014). Authors noted that isoprene emission in response to drought stress largely depends on the strategies adopted by plants to cope with the scarcity of water, and may not be a good proxy of its leaf internal concentration. For example, Martinez-Sancho et al. showed that contrasting xylem anatomy and hydraulics in the evergreen isohydric *Pinus sylvestris* and in the deciduous anisohydric broadleaf *Quercus robur*, yielded different stomatal sensitivity to dehydration. Oak adjusted vessel size to maintain high stomatal conductance and carbon uptake, while avoiding embolism under increasing aridity during drought. Pine, instead, relied more on stomatal closure to prevent excessive water loss. Indeed, these contrasting stomatal behaviors determine the ratio between the biosynthesis and the emission of isoprenoids and open new questions about their protective roles under drought stress of increasing severity. The effect of moderate drought on volatiles emission by two ecotypes of *Arundo donax*, a fast-growing, isoprene emitter has been evaluated by Haworth et al. They investigated the methyl-erythritol and the methionine pathways, linked to carotenoid/isoprene and glutathione biosynthesis, respectively. Authors suggested that both cycles are independent chloroplastic pathways, both aimed at preserving photosynthetic capacity and enhancing tolerance to moderate drought, and no trade-off occurs between the two pathways.

Solid pollutants from human activities include heavy metals, black carbon, some polycyclic aromatic hydrocarbons, and the much less explored nanoparticles. Lead is one of the most abundant pollutants, readily absorbed through the root system of edible plants and entering the food-chain, with adverse consequences on public health worldwide. Wang Y. et al. examined the mechanism underlying root proteome in response to Pb in radish. Key enzymes in response to Pb relate with glycolysis, citric acid cycle, and the scavenging of reactive oxygen species. The response of floating and submerged leaves of *Potamogeton nodosus* to Ag toxicity was examined by Shabnam et al.. They showed that the antioxidant machinery of floating leaves is responsible for their superior Ag tolerance as compared to submerged leaves, and provide new insights to assist effective bioremediation of water bodies. Besides, the rising release of nanoparticles to the environment from household, agricultural, industrial, and healthcare products pose urgent need to understand their effect on plant growth, development, and physiology. Marslin et al. reviewed this topic and found that exposition to nanoparticles induces a ROS signaling that activate secondary metabolic pathways. Due to their sensitivity to environmental disturbances, including air pollution, and their capillary distribution in cities, urban trees can be effective low-cost indicators of air quality in urban sites. Ferreira et al., using a combination of bioindication and geostatistical methods, showed that the analysis of tree bark is a precise, still inexpensive tool for assessing the spatial distribution of airborne solid pollutants within a city as well as citizens' health risking areas lacking traditional pollution monitoring networks. Electromagnetic pollution is a novel pressure imposed by human activities impose to plant communities, still research has only focused on its effects on animals. Sztafrowski et al. analyzed the effect of magnetic fields on gene expression in *Solanum tuberosum* and *A. thaliana*, to evaluate if electromagnetic fields trigger similar effects on gene transcription as those observed in humans.

Understanding plant response to the unprecedented challenges associated to rapid climate change has been the aim of this Research Topic. Such knowledge is urgently needed to develop effective conservation strategies, and to set-up long-term monitoring networks aimed at observing the impact of multiple stress pressures on forest ecosystems. Suitable physiological indicators for this purpose, such as carbon isotope composition and photosystem II photochemistry efficiency, are well described in Bussotti and Pollastrini. Biochemical markers can also assist monitoring and guide restoration programs. This may be the case of methionine, suggested by Taïbi et al. as a suitable marker for screening drought tolerant ecotypes of *Pinus halepensis*. We have provided information regarding plants responses to abiotic and biotic stresses from multiple organization levels (molecular, cell, organism, and ecosystem) giving new evidence that may help to better understand how plants will cope with stress factors and novel pressures imposed by the global warming and anthropogenic pressure. However, this topic is wide, which and has been highlighted in other research topics in Frontiers in Plant Science, namely “water stress responses in plants,” “mechanisms of abiotic stress responses and tolerance in plants,” or “photosynthesis within changing climate conditions.” These three are only examples of the 90 research topics published regarding “climate change” or of the 450 concerning “plant responses to stress.” Nonetheless, further experimentation is urgently needed to understand and predict the interactions that underpin the special capacity of plants to fine-tune their physiology and (secondary) metabolism to successfully respond to the severe challenges imposed by a rapidly changing climate, particularly in most fragile ecosystems worldwide. This requires approaches at different levels of scale, using bottom-up as well as top-down approaches, not only to breed “resistant” crops and/or selected varieties, but also to additionally develop strategies for promoting both biodiversity conservation and ecosystem functionality.

## Author contributions

All authors listed have made a substantial, direct and intellectual contribution to the work, and approved it for publication.

### Conflict of interest statement

The authors declare that the research was conducted in the absence of any commercial or financial relationships that could be construed as a potential conflict of interest.
